# Association of *Plasmodium berghei* With the Apical Domain of Hepatocytes Is Necessary for the Parasite's Liver Stage Development

**DOI:** 10.3389/fcimb.2019.00451

**Published:** 2020-01-17

**Authors:** Lakshmi Balasubramanian, Vanessa Zuzarte-Luís, Tabish Syed, Debakshi Mullick, Saptarathi Deb, Harish Ranga-Prasad, Jana Meissner, Ana Almeida, Tobias Furstenhaupt, Kaleem Siddiqi, Miguel Prudêncio, Cecilia M. P. Rodrigues, Maria Mota, Varadharajan Sundaramurthy

**Affiliations:** ^1^National Center for Biological Sciences, Bangalore, India; ^2^Instituto de Medicina Molecular, Faculdade de Medicina, Universidade de Lisboa, Lisbon, Portugal; ^3^School of Computer Science and Centre for Intelligent Machines, McGill University, Montreal, QC, Canada; ^4^Max Planck Institute of Molecular Cell Biology and Genetics, Dresden, Germany; ^5^Research Institute for Medicines, University of Lisbon, Lisbon, Portugal

**Keywords:** plasmodium liver stage, hepatocyte polarity, ultrastructure, 3D image analysis, liver tissue, bile canaliculi

## Abstract

*Plasmodium* parasites undergo a dramatic transformation during the liver stage of their life cycle, amplifying over 10,000-fold inside infected hepatocytes within a few days. Such a rapid growth requires large-scale interactions with, and manipulations of, host cell functions. Whereas hepatocyte polarity is well-known to be critical for liver function, little is presently known about its involvement during the liver stage of *Plasmodium* development. Apical domains of hepatocytes are critical components of their polarity machinery and constitute the bile canalicular network, which is central to liver function. Here, we employed high resolution 3-D imaging and advanced image analysis of *Plasmodium*-infected liver tissues to show that the parasite associates preferentially with the apical domain of hepatocytes and induces alterations in the organization of these regions, resulting in localized changes in the bile canalicular architecture in the liver tissue. Pharmacological perturbation of the bile canalicular network by modulation of AMPK activity reduces the parasite's association with bile canaliculi and arrests the parasite development. Our findings using *Plasmodium*-infected liver tissues reveal a host-*Plasmodium* interaction at the level of liver tissue organization. We demonstrate for the first time a role for bile canaliculi, a central component of the hepatocyte polarity machinery, during the liver stage of *Plasmodium* development.

## Introduction

*Plasmodium* parasites undergo a dramatic amplification during the liver stage of their life cycle, when an individual sporozoite infecting a hepatocyte multiplies inside a parasitophorous vacuole (PV) to produce several thousand infective merozoites (Prudencio et al., 2006; Vaughan and Kappe, 2017). This rapid intra-hepatic growth requires extensive networking and interactions with the host's liver cells, at both the sub-cellular and molecular levels (Agop-Nersesian et al., 2018; Nyboer et al., 2018). Pioneering ultrastructure studies employing sporozoite infection of a restricted area of rat livers have suggested extensive interactions of the PV membrane (PVM) with different host organelles during parasite development (Meis et al., 1981, 1983a,b; Shin et al., 1982). Some of these interactions, such as those with the autophagosome, late endosomes, lysosomes and the endoplasmic reticulum, play central roles in nutrient acquisition and immune evasion, and are necessary for supporting parasite development inside hepatocytes (Bano et al., 2007; Lopes da Silva et al., 2012; Thieleke-Matos et al., 2016; Coppens, 2017; Evans et al., 2018).

One of the defining features of hepatocyte function is their unique polarity. Unlike a columnar epithelial cell, where the entire surface facing a luminal cavity is apical, hepatocytes have apical domains spanning the cell as “bands” that connect in 3 dimensions to form the highly ramified bile canalicular (BC) network. This network constitutes the first level of branching in the complex bile duct tree, which eventually drains into the gall bladder (Elias, 1949; Treyer and Musch, 2013; Gissen and Arias, 2015). The geometry of the bile duct tree plays a crucial role in the production, flux, and storage of bile (Meyer et al., 2017). Correct polarization of hepatocytes and organization of the apical domains are essential for bile secretion and flow (Arias et al., 1993; Turumin et al., 2013), while loss of polarity is associated with several liver diseases (Gissen and Arias, 2015). The velocity of the bile flow depends on the secretion of bile by hepatocytes into their apical domain and on the geometric features of the bile canalicular network (Meyer et al., 2017). Thus, apical domain organization is a critical aspect of hepatocyte function. Whether these processes are involved in the liver stage of *Plasmodium* infection is not presently known.

The critical role of polarity for hepatocyte function, and the specific tropism of *Plasmodium* sporozoites for hepatocytes, motivated us to explore the connection between hepatocyte polarity and *Plasmodium* development during the liver stage of infection. Hepatoma cells in 2D culture systems typically lose the characteristic hepatocyte polarity (Treyer and Musch, 2013; Musch, 2014; Gissen and Arias, 2015), and are hence unsuitable models to address this question. We therefore departed from the classical approach, by studying the development of the *Plasmodium* parasite in its native three-dimensional tissue environment. To that end, we employed high resolution 3D imaging and advanced and customized quantitative image analysis of infected liver tissues to show that the parasite makes preferential contacts with the hepatocyte's apical domain during its development in the liver. Furthermore, these apical domains are themselves re-organized during the liver stage of *Plasmodium* development, resulting in localized alterations in bile canalicular architecture. Finally, we show that pharmacological manipulation of hepatocyte polarity alters the bile canalicular architecture, preventing the contact of the hepatocyte's apical domain with the parasite vacuole membrane and arresting its development.

## Materials and Methods

### Mice and *Plasmodium berghei* Liver Infection

All mice used in this study were C57BL/6J mice purchased from Charles River Laboratories (L'Arbresle, France), housed in the facilities of the Instituto de Medicina Molecular and allowed free access to water and food. Infections were performed using a GFP-expressing *P. berghei* ANKA parasite line (259cl2). Mice were infected by intravenous injection of 10^5^ sporozoites obtained through dissection of the salivary glands of infected female *Anopheles stephensi* mosquitoes bred at the Instituto de Medicina Molecular. Hepatic infection was determined by microscopy analysis of liver sections or by quantitative RT-PCR amplification of *Plasmodium* 18S rRNA, at specific times of infection. Salicylate (Calbiochem 71945) was administered by intraperitoneal injection of 300 mg/kg in NaCl 0.9% at 2 and 24 h post infection (hpi). All experiments were approved by the animal ethics committee at Instituto de Medicina Molecular and performed in strict compliance with the guidelines of National and European regulations.

### Bile Acid Analysis

Bile acids were extracted from 5 μl of gallbladder bile by liquid-solid extraction using Sep Pak C18 cartridges (Waters, Milford, MA, USA). The samples were diluted in 0.1 M Tris HCl, pH 9.0, passed through activated cartridges, eluted with methanol, taken to dryness under an N2 stream, re-dissolved in methanol and saline (1:10, v/v) and then subjected to an enzymatic colorimetric assay. The total volume of bile acids in gallbladder bile and serum (150–200 μl) was determined using the 5th Generation Enzymatic Colorimetric RX Series kit (Randox Laboratories Ltd, Crumlin, UK) and the HORIBA Medical Clinical Chemistry Analysis Pentra C200 (Kyoto, Japan).

### Fixation, Staining, and Immunofluorescence Microscopy Analysis of Liver Tissues

At selected time points after sporozoite infection, livers were perfused through the portal vein with 4% PFA and further fixed by overnight incubation in 4% PFA at 4°C. One-hundred micrometer thick sections were prepared from perfusion-fixed infected mouse liver using a vibratome (Leica VT1200S). The sections were immuno-stained using standard methods (Meyer et al., 2017) to mark the parasitophorous vacuole membrane (PVM) using a UIS4 antibody (Sicgen, dilution 1:500), and the apical domain using a CD13 antibody (Novus biologicals, dilution 1:200). Nuclei and cell boundaries were visualized using DAPI (Invitrogen) and Phalloidin (Invitrogen, dilution 1:250), respectively. Briefly, floating sections were permeabilized by incubation in 0.5% Triton X-100 in PBS for 60 min, washed thrice in 0.2% fish gelatin, 300 mM NaCl and 0.3% Triton-X100 in PBS, and then incubated in a primary antibody in the same buffer for two overnight incubations with the section flipped once in between. Sections were then washed extensively (5 changes of 15 min each) followed by two overnight incubations with a secondary antibody in the same buffer, with the section flipped after 1 day. Typically DAPI and Phalloidin were added along with this mix. The stained sections were cleared by SeeDB using standard methods (Ke et al., 2013). Briefly, immunostained sections were washed 5 × 15 min with 0.3% Triton X-100, followed by a 3 × 1 min wash in PBS. They were then incubated in progressively increasing concentrations of fructose solutions (25, 50, 75, and 100%) for 8–12 h at room temperature for each concentration of fructose, until the floating sections sank to the bottom. Finally, the sections were incubated with SeeDB overnight at room temperature and mounted on a glass slide in SeeDB with a coverslip of 0.17 ± 0.005 mm thickness. The slides were imaged using a Zeiss LSM780 confocal microscope using a 63x objective, NA 1.4 with four lasers, 405 nm to detect DAPI, 488 nm for CD13, 568 nm for UIS4 and 647 nm for Phalloidin-647. The images were typically acquired up to a z-depth of 75–90 μm with a voxel size of either 0.3 or 0.5 μm^3^, with pinhole set to 1 Airy unit. Typically, the x-y area covered a field of 134.95 μm^2^.

### Image Analysis

Image analysis was carried out using either CellProfiler (Carpenter et al., 2006; Lamprecht et al., 2007; Kamentsky et al., 2011) or customized macros in Fiji (Schindelin et al., 2012). The Fiji macros were semi-automated and were used to segment the cellular organelles from the infected liver tissue and to estimate the tubular and network features of the bile canaliculi. Details on the macros and the processing steps are provided in Supplementary Material. A fully automated geometric flow-based segmentation method (Vasilevskiy and Siddiqi, 2002) using customized Matlab codes was also employed to independently segment the BC and PVM in 3D, and the results were compared against those obtained using intensity threshold based segmentation with manual interaction.

### EM Sample Preparation

One hundred micrometer thick vibratome sections were cut from perfusion-fixed (2% formaldehyde, 2.5% glutaraldyhde in PBS) *P. berghei*-infected livers (Leica, Vienna 1200S) and stored in 1xPBS at 4°C. A Zeiss Axioplan2 upright epifluorescence microscope with an RTSPOT monochrome camera was used to locate the parasites within the sections. The region of interest containing the parasite was cut with a razor blade and placed into 3 × 0.1 mm gold-coated sample carriers (Leica) for high pressure freezing. The carrier was filled with 20% BSA in a 0.1 M phosphate buffer for cryo protection. The liver tissue pieces were high pressure frozen (Leica EM ICE) and freeze substituted (Leica EM/AFS2) in 1% osmium tetroxide, 0.1% uranyl acetate in acetone, rinsed in acetone at RT and then flat-embedded in epoxy resin (EMBed 812, EMS). Three hundred or seventy nanometer thick sections were cut from the polymerized tissue blocks for tomogram and ultrastructure imaging, respectively. The grids were stained with uranyl acetate and lead citrate according to standard protocols. Gold fiducials were added prior to imaging for the tomograms.

Transmission Electron Tomography was performed using a Tecnai TF30 G2 FEG-TEM (Thermo Fisher Scientific Electron Microscopy, Hillsboro, Oregon, USA) with a Fischione 2040 Dual-Axis Tomography Holder (Fischione Instruments, Pennsylvania, USA). All images were acquired on a Gatan UltraScan 1,000 CCD (Gatan, Pleasanton, California, USA) at 2,048 × 2,048 pixels at a microscope magnification of 4,700. The acceleration voltage was 300 kV with 4,500 V extraction voltage at the Field Effect Gun at spot size 1 and gunlens 1. Dual axis tomography was carried out by taking one image every degree, for a tilt range between 120 and 130 degrees, with the program SerialEM (Mastronarde, 2005), with exposure times between 0.8 and 1.3 s. The tomographic reconstruction was performed by weighted back-projection with the IMOD software package (Kremer et al., 1996; Mastronarde, 1997). The same software was used for visualization and analysis, including 3D rendering.

## Results

**Figure 1:**
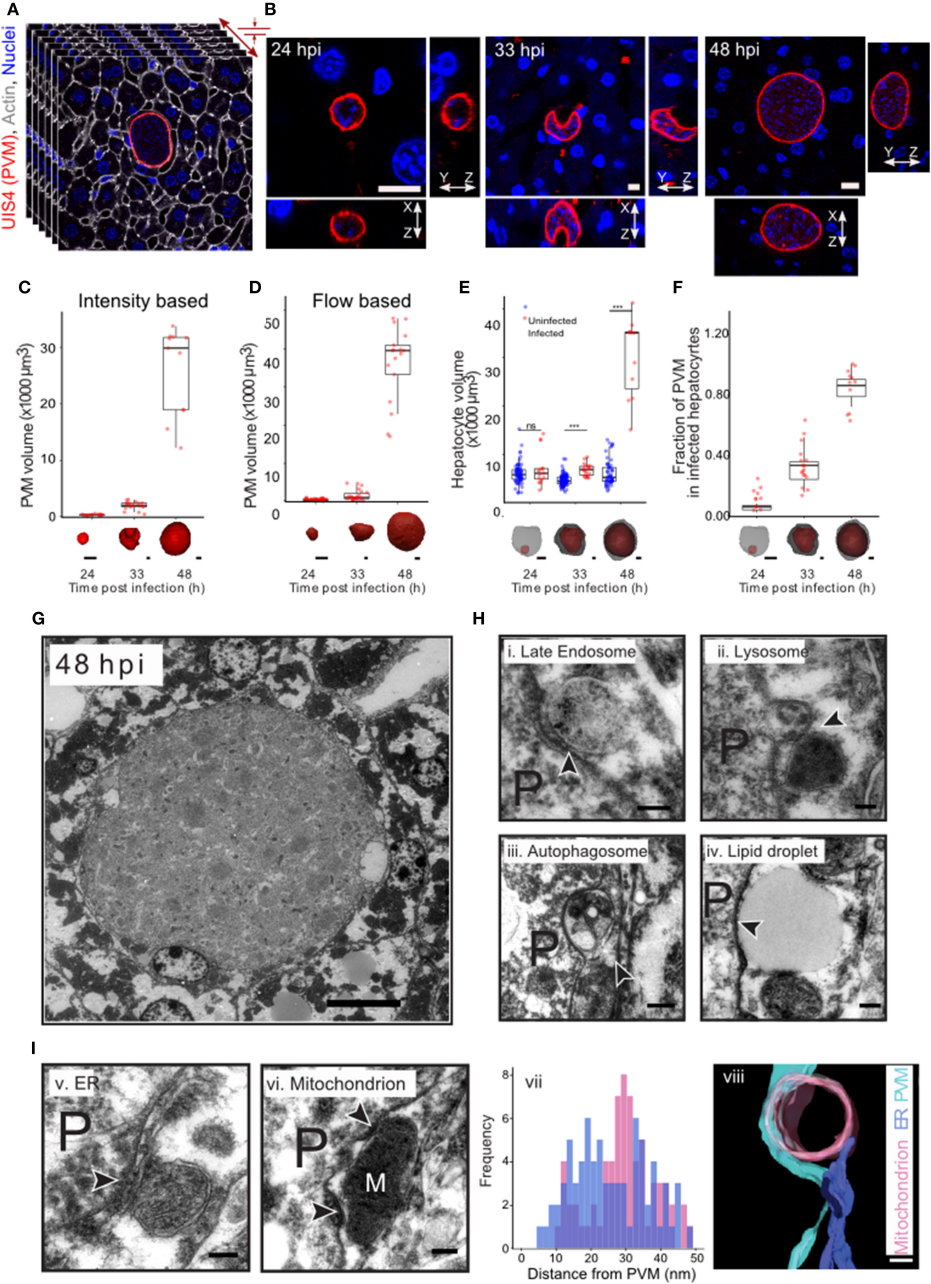
Plasmodium liver stage development *in vivo* in 3d. **(A)** 3D imaging and analysis of *Plasmodium berghei* development during the liver stage of infection. One hundred μm liver sections from *P. berghei* infected mice were sectioned and stained for PVM using an anti-UIS4 antibody. Nuclei and cell membranes are visualized by DAPI and Phalloidin staining, respectively. Imaging was carried out using confocal microscopy at a resolution of 0.3 μm voxel. **(B)** Representative images from *P. berghei*-infected liver sections collected 24, 33, and 48 hpi, showing the PVM and nucleus in x-y, x-z, and y-z views. The scale bar is 10 μm. **(C)** Quantification of volume changes in the parasitophorous vacuole during *P. berghei* development *in vivo*. Images from B were segmented using an intensity thresholding method. **(D)** Quantification of volume changes in the parasitophorous vacuole during *P. berghei* development *in vivo*. Images from B were segmented using an automated geometric flow based segmentation method. **(E)** Quantification of volume changes at the indicated time points post infection in the infected and nearby uninfected hepatocytes. ^***^denote a *p* < 0.001 by Student's *t*-test. **(F)** Proportion of host cell volume occupied by the parasite during liver stage development *in vivo*. For **(C–F)**, a representative image of the model for the segmented parasite (red) and the host hepatocyte (gray) in 3d, for the time points indicated, is shown along the x-axis. The scale bar is 10 μm. **(G)** Ultrastructure of *P. berghei*-infected cell at 48 hpi *in vivo*. The scale bar is 10 μm. **(H)** Selected regions from a *P. berghei* infected hepatocyte *in vivo* showing close association of the PVM with late endosome (i), lysosome (ii), autophagosome (iii), and a lipid droplet (iv). **(I)** Selected regions from a *P. berghei* infected hepatocyte *in vivo* showing close association of the PVM with ER (v) and a mitochondrion (vi). Distance distribution of PVM with ER (purple) and mitochondria (pink) (vii). Data is from at least 5 infected cells. (viii) shows a representative tomogram reconstruction of a close interaction of the PVM (cyan) with a mitochondrion (pink) and ER (purple). For **(H,I)**, P denotes the parasite and the arrowheads point to the apposition of PVM with the indicated organelles. The scale bar is 200 nm.

In order to comprehensively characterize hepatic *P. berghei* development in its native liver tissue environment, 100 μm-thick sections of mouse livers collected 24, 33, and 48 h post-infection (hpi) were immunostained with UIS4 to mark the PVM and stained with DAPI and phalloidin, to label the nuclei and host cell boundaries, respectively. These time points were selected to specifically study *Plasmodium* hepatic schizogony following the successful completion of the initial invasion and dedifferentiation steps. For the purpose of this study, we consider the selected time points to correspond roughly to “early,” “mid,” and “late” stages of parasite schizogony. We performed high resolution 3D imaging at isometric sub-micron resolution (Figures 1A,B, Movie S1A), and acquired 3D images of the entire volume of the infected cell. The PV volume was quantified by segmenting the vacuole in 3D using an intensity thresholding method and compared across the different time points. Our results show that parasite growth *in vivo* is not linear, displaying only a modest increase in PV volume between 24 and 33 hpi, followed by a steep increase between 33 and 48 hpi (Figure 1C), showing that the period between 33 and 48 hpi marks a phase of rapid parasite development *in vivo*. We modified a flow-based segmentation method (Vasilevskiy and Siddiqi, 2002) to establish an automated method for object segmentation in 3D on this dataset (Movies S1C,S1D, Figure 1D). Next, we measured the changes in hepatocyte volume accompanying parasite growth at these time points, by segmenting infected and uninfected hepatocytes from the same tissue (Movie S1B). Our results show that the volume of the infected cell was comparable to that of non-infected cells at 24 hpi, but showed a modest increase at 33 hpi (Figure 1E). Most strikingly, at 48 hpi, the volume of the infected cell increased 6–8-fold relative to that of the non-infected cells (Figure 1E). The fraction of the cell volume occupied by the parasite increased from 10 to 35% between 24 and 33 hpi, and reached almost 90% by 48 hpi (Figure 1F), in agreement with the dramatic parasite growth observed *in vivo*. The large increase in the volume of the infected hepatocyte observed at late stages of infection is likely to influence neighboring cells. Given that the number of uninfected cells far outnumbers that of infected cells, these data suggest that the intrahepatic development of *Plasmodium* parasites likely results in highly localized changes to liver tissue, specifically around the infected cells.

The striking changes observed in intracellular *Plasmodium* development during the liver stage of infection likely necessitate extensive interactions with the host cells at a sub-cellular level. In order to systematically explore these interactions during parasite development *in vivo*, we optimized methods for ultrastructural observation of infected cells in the liver tissue at 33 and 48 hpi (Figures 1G,H). We observed close interaction of PVM with late endosomes, lysosomes, autophagosomes, lipid droplet, ER and mitochondria (Figures 1H,I). Late endosomes and lysosomes were identified based on their characteristic morphology (Zeigerer et al., 2012), autophagosomes were distinguished by the presence of double membrane, and the ER was identified based on its characteristic tubular structure. In particular, contacts with mitochondria and ER were proximal and frequent, as shown by a representative tomogram (Figure 1I,viii) and by distance distribution plots (Figure 1I,vii), respectively. Thus, extensive contacts with diverse host cellular organelles are established and maintained during liver stage infection *in vivo*. Collectively, these observations exemplify the interactions occurring between *Plasmodium* and the host cell during the parasite's liver stage development.

**Figure 2:**
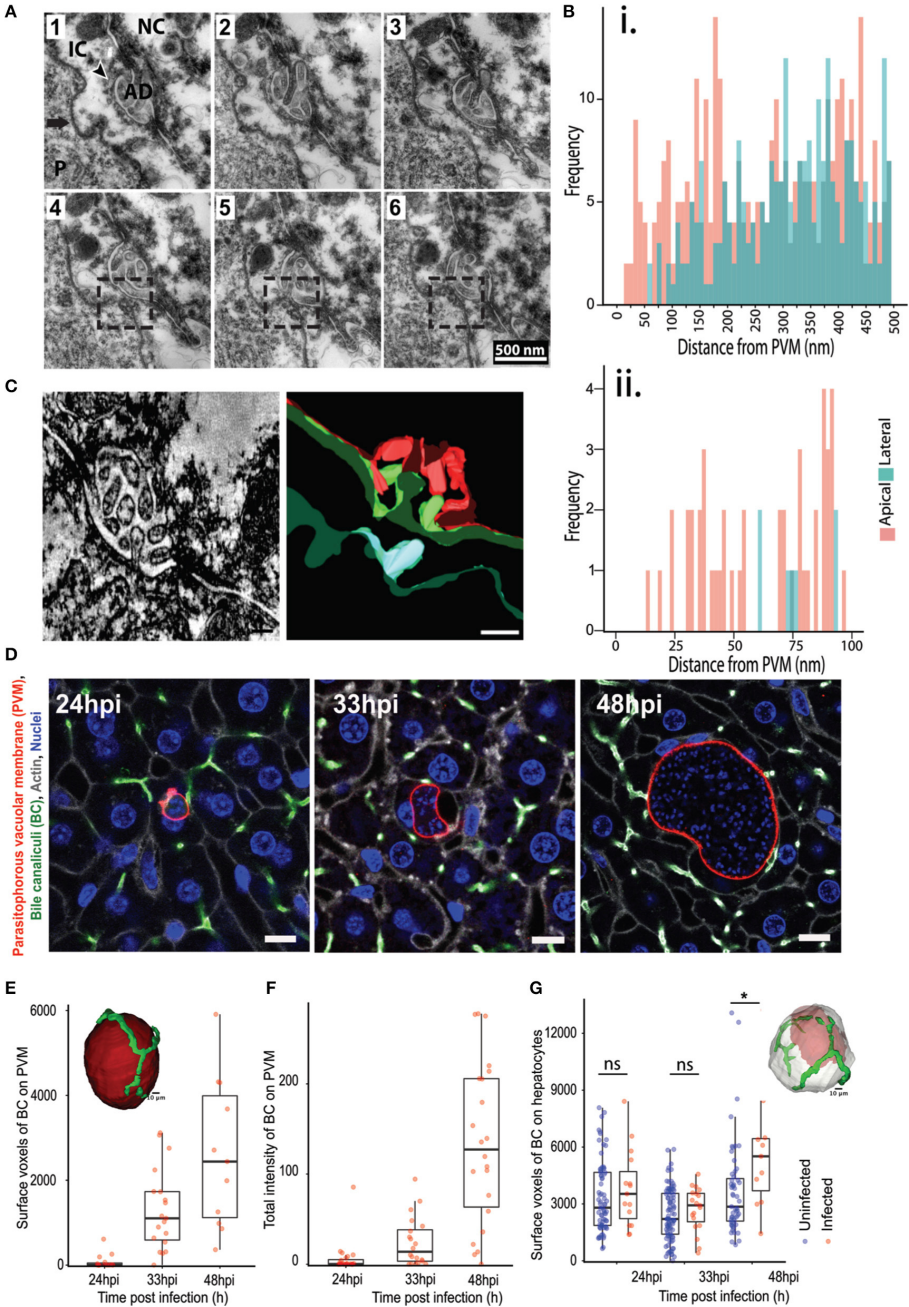
Association of *P. berghei* with hepatocyte apical domains during liver stage development. **(A)** Serial EM sections showing the PVM in close proximity to the apical domains of the hepatocyte plasma membrane. The sections are 70 nm apart, with the boxed region highlighting the juxtaposition of the PVM with the apical region. The arrow points to the PVM, with the arrowhead showing the apical domain (AD). P indicates the parasite. **(B)** Quantification of the distance between the PVM and apical (pink) or basolateral (green) regions of the hepatocyte plasma membrane. Data is combined from distance measurements from at least five infected cells. (ii) highlights a region from (i) showing spatial proximity of the PVM to apical domains. **(C)** A section of a tomogram of the PVM associated with an apical region (left) and the corresponding reconstruction (right). The PVM is in cyan, the plasma membrane of the infected hepatocyte in green, and the plasma membrane of the adjacent non-infected hepatocyte in red. **(D)** Representative confocal images of infected liver sections at 24, 33, and 48 hpi, showing PVM (red), bile canaliculi (green), cell boundaries (gray), and nuclei (blue). The UIS4 and CD13 antibodies were used to mark the PVM and apical domains, respectively. The scale bar is 10 μm. **(E)** Quantification of surface voxels of the apical domain (CD13) on the PVM at different time points post infection. The inset figure shows the segmented model of apical domains (green) in proximity with the PVM (red). The scale bar is 10 μm. **(F)** Quantification of the total intensity of CD13 (apical domain/bile canaliculi marker) on the PVM at different time points post infection. The inset shows a schematic of the pipeline used for quantitative analysis. **(G)** Quantification of the alteration in hepatocyte polarity during *P. berghei* infection *in vivo*. Surface voxels of the apical domain on the infected hepatocyte surface in comparison with an uninfected hepatocyte at different time points post infection are shown. n.s and ^*^denote *p* values that are non-significant and < 0.01 respectively, based on Student's *t*-test. The inset shows a model of 3D segmentation of an infected hepatocyte (gray) containing a parasite (red), with the apical domains (green).

In addition, we also observed that the PVM is often in close contact with the apical domain of the hepatocyte, as illustrated by EM imaging of 70 nm thick serial liver sections (Figure 2A). Apical membranes are defined by the electron dense tight junction and the characteristic involutions of the bile canaliculi (BC) (Goldblatt and Gunning, 1984). In order to study this association further, we measured the distance from regularly spaced points in the PVM to the closest point in the hepatocyte plasma membrane, and further categorized the hepatocyte membrane as either apical or basolateral. Frequency plots of these measurements from five infected cells using ultrastructural EM show that the PVM tends to be closer to the apical membranes than the baso-lateral ones (Figure 2B). We confirmed this result by electron microscopy tomogram analysis of a 300 nm thick section of an infected cell (Figure 2C, Movie S2A). EM analysis of a limited number of infected cells suggested an interaction between the PVM and the host hepatocyte's apical domain. Apical domains are a central feature of the unique hepatocyte polarity, as apical regions from the plasma membrane of adjacent cells connect in 3 dimensions to form the bile canaliculi, which represents the first level of organization in the complex 3 dimensional bile network geometry (Arias et al., 1993; Treyer and Musch, 2013; Gissen and Arias, 2015).

We sought to confirm this potentially novel interaction by an independent method and substantiate it with quantitation from several infected cells. To this end, high resolution 3D imaging and image analysis of infected mouse liver tissues were performed using customized platforms on infected liver sections stained for the apical domain marker CD13 and imaged using confocal microscopy at isometric voxels size of 0.3 μm (Figure 2D). Images were reconstructed in 3D to visualize the PVM in conjunction with the apical domain of the infected hepatocytes. Two independent customized image analysis workflows were employed to analyze the PVM-BC association (Figures S2, S3). The first method employed customized macros in Fiji to subtract the BC image from the PVM in 3D, in order to identify and quantify the total intensity of the BC regions overlapping with the PVM (method outlined in Figure S2). The surface voxels of BC on the PVM were quantified as a measure of the PVM-BC association. These voxels represent parts of the BC that are in direct contact with the PVM; values higher than zero denote juxtaposition and are proportional to the extent of the overlap. The second method employed CellProfiler to segment the PVM and BC in individual slices, and extract the intensity of BC that specifically overlaps the BC. The intensity values, extracted for each slice, were added over all the 3D stacks to generate a single value that represents the total fluorescence intensity of PVM on BC for the entire 3D volume of a given PVM. Both methods yielded consistent results (Figures 2E,F), showing that the PVM-BC associations are formed early during parasite development and expand over time, to mirror the growth pattern of the parasite development *in vivo*. The appositions at 48 hpi could be due to the PVM occupying up to 90% of hepatocyte volume so that such contacts are a consequence of the large parasite size. For this reason, we also quantified associations at 24 and 33 hpi, when both the infected cell size and the PVM size are not likely to be confounding factors. Our analysis at 24 hpi shows that, even at this time point, when the parasite is about the size of the hepatocyte nucleus, the PVM is in close proximity to the BC (denoted by non-zero values) and these associations increase considerably at 33 hpi (Figures 2E,F), suggesting that the PVM has a spatial preference within the infected cell as the infection proceeds. We tested if the apical domain contact scales with the PVM volume using scatter plot analysis. The results (Figure S5) show a positive correlation at 33 hpi, but not at 24 or 48 hpi, suggesting that the apical domain contact scales with parasite volume during the expansion phase of the parasite. At 24 hpi it is likely that the contacts are starting to form and at 48 hpi, the correlation could be lost due to the large volume the parasite attains. Finally, we tested the specificity of the PVM-BC association by using the flow based method to assess the chances of a randomly placed sphere of volume equivalent to the PVM to form similar associations with the BC network (Supplementary Method, Figure S6). The results show that an inert bead randomly placed at a distance “r” from the BC network would be more likely to not intersect the BC network than to intersect it.

We then assessed whether the close association of the PVM with BC, together with the massive increase in the parasite and host cell volume, result in alterations in the apical domain organization around the infected cell. To this end, we quantified the total apical domain of the infected cells and compared it with that of the uninfected cells. In order to preclude any proximity effects on uninfected cells due to close positioning with infected cells, we selected uninfected cells at least two cell layers away from the infected cells for this analysis. Our data show that, at 48 hpi, there is a significant increase in the apical surface area of the infected cells compared to uninfected cells (Figure 2G). These changes may be due to the massive increase seen in the infected cell volume at this time point. Nevertheless, since apical domains from adjacent cells connect in 3D to form the bile canaliculi, this result suggests that localized alterations in the BC network geometry occurs during the liver stage of *Plasmodium* development.

**Figure 3:**
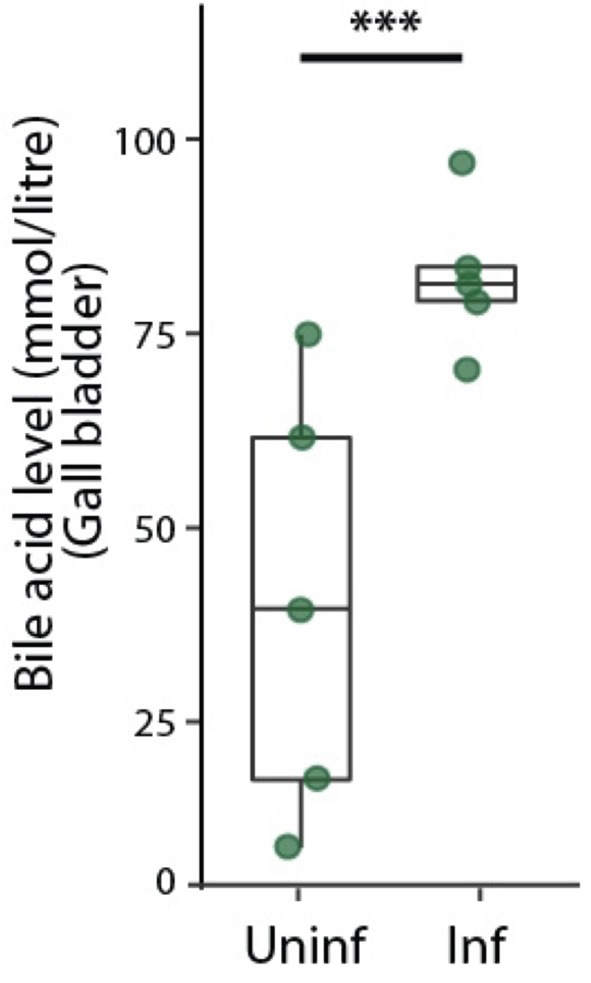
Infected mice show higher levels of bile acid levels in the gallbladder. Total bile acid levels in gallbladders of *P. berghei*-infected and uninfected mice at 48 hpi. Result representative of two biological replicates. ^***^denotes *p* < 0.001 from Student's *t*-test.

Bile acids are secreted into bile canaliculi through transporters localized on the apical domains of hepatocytes and are transported through the bile canalicular network to eventually drain into the gallbladder (Turumin et al., 2013). We reasoned that alterations in bile canalicular network could impact this process and possibly reflect in the gallbladder bile acid levels. To test this, we measured bile acid levels in the gallbladder during the liver stage of infection. Surprisingly, we found that the bile acid levels are significantly higher in infected mice than in uninfected controls (Figure 3). Moreover, bile acid levels increased with increasing dose of sporozoites injected, correlating with the ensuing hepatic parasite burden at 40 hpi (Figure S7). Since biliary secretion is a major function of liver that requires hepatocyte polarization, this result suggest that Plasmodium liver stage infections impact liver function, possibly by impacting the apical domain organization.

**Figure 4:**
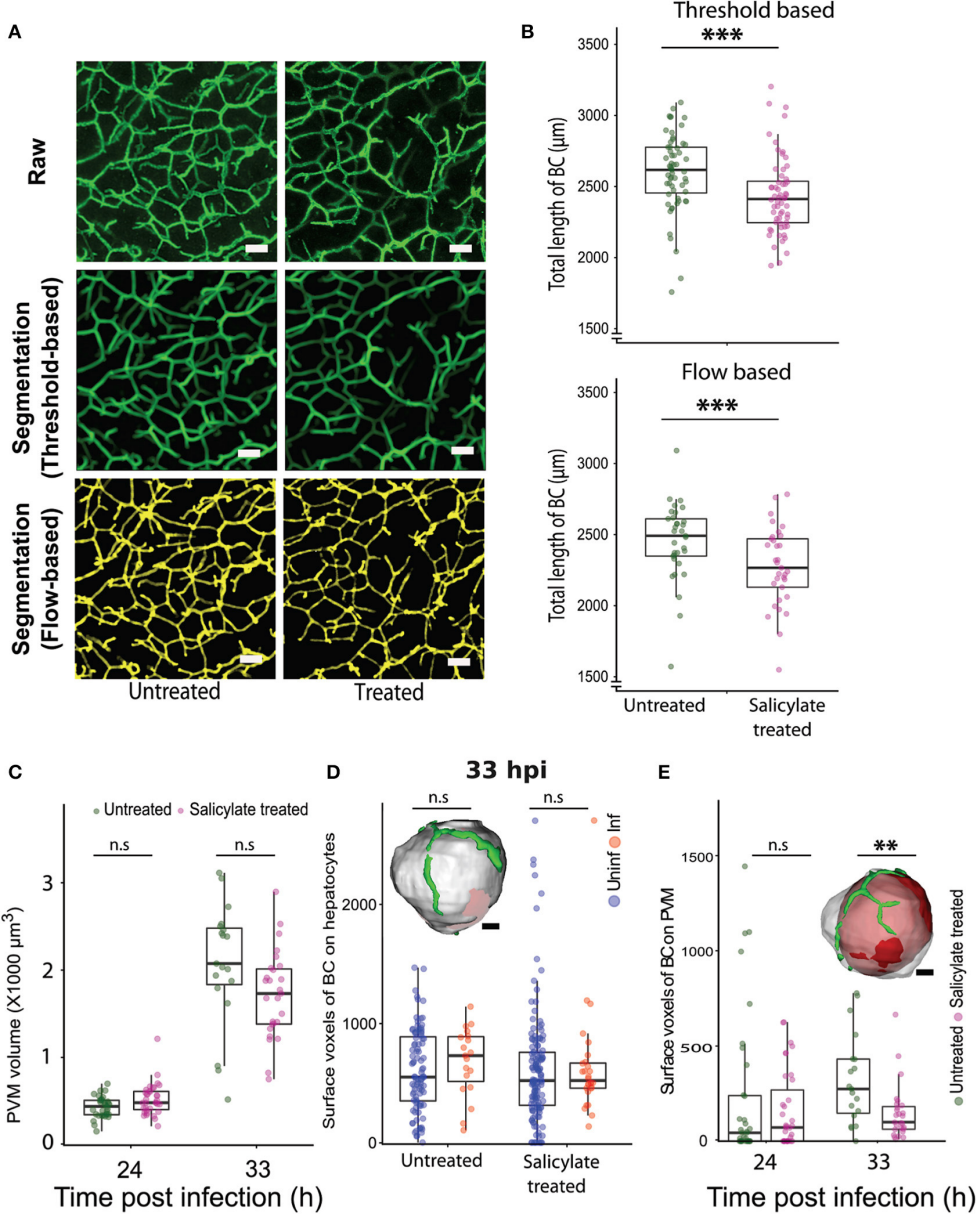
Pharmacological modulation of hepatocyte polarity *in vivo* arrests *P. berghei* development in liver tissues. **(A)** Representative images of maximum intensity projections of liver sections from untreated and salicylate-treated mice, stained with the apical domain marker CD13. One hundred μm thick liver sections were imaged with a voxel size of 0.5 μm^3^. The scale bar is 10 μm. The top panel shows the raw image, the middle panel shows segmentation based on thresholding and the lower panel shows segmentation using an automated flow based method. **(B)** Quantification of total length of bile canaliculi (BC) between untreated and Salicylate treated mice. The top and bottom panels show quantifications from threshold based and flow based segmentation, respectively. **(C)** PVM volume changes upon salicylate treatment at 24 and 33 hpi. n.s. denotes the differences between treated and untreated conditions that are not statistically significant at these time points. **(D)** A comparison of surface voxels of bile canaliculi (BC) on hepatocytes between uninfected and infected hepatocytes, with and without salicylate treatment. The inset shows a representative segmented model of infected hepatocyte (gray) and bile canaliculi (green). The scale bar is 10 μm. n.s denotes the differences are not statistically significant. **(E)** Surface voxels of bile canaliculi on the PVM between Salicylate treated and untreated conditions. The inset shows a representative segmented model of PVM (red) within an infected hepatocyte (gray) with surrounding bile canaliculi (green). The scale bar is 10 μm. **(A–E)** Are representative of two biological replicates, n.s. denotes the differences are not statistically significant, ^**^ and ^***^denote *p* < 0.01 and < 0.001, respectively. Statistical significance is assessed using Student's *t*-test.

Next, we explored the relevance of the localized alteration in apical domain organization for *Plasmodium* development in the liver. The AMPK pathway is a key regulator of tight junction formation and apical trafficking, thereby influencing apical domain organization and hepatocyte polarity (Treyer and Musch, 2013; Musch, 2014; Gissen and Arias, 2015). Hence, we wondered whether pharmacological modulation of the AMPK pathway would alter bile canalicular geometry *in vivo*. To evaluate this, mice were treated with a well-known AMPK modulator, salicylate (Hawley et al., 2012), and 100 μm-thick liver sections were collected from both salicylate-treated and untreated control mice, and stained for the bile canalicular marker, CD13. The bile canalicular network was then imaged at isometric voxels at 0.5 μm resolution. The images were segmented in 3D using intensity-based thresholding as well as automated flow-based methods (Movies S4A, S4B, Figure 4A), and the total length of the BC network was calculated for the salicylate-treated and untreated conditions. The result shows significant alterations in the bile canalicular network upon salicylate treatment (Figure 4B), confirming that BC geometry is indeed altered *in vivo* upon AMPK activation. We next assessed the effect of pharmacological activation of AMPK on *P. berghei* hepatic development *in vivo*. To this end, parasite size in the livers of salicylate-treated and untreated control mice was quantified at 24, 33, and 48 hpi. While no significant differences in parasite volume were observed upon salicylate treatment at 24 and 33 hpi (Figure 4C), parasite growth was significantly attenuated in salicylate-treated mouse livers at 48 hpi (Figure S8), in agreement with a previous report (Ruivo et al., 2016). This suggests that salicylate treatment does not have an effect on the initial growth phase of the parasite but impacts its subsequent rapid expansion phase. Our data (Figures 2E,F) shows that the association of PVM with the BC mirrors the parasite growth dynamics *in vivo*. Since the parasite volume is not affected up to 33 hpi upon salicylate treatment but is arrested at 48 hpi, we hypothesized that salicylate-mediated global alteration in BC geometry locally affects the PVM contact with the apical domain in the infected cell. Since the salicylate-mediated alteration in BC geometry is global, whereas parasite-mediated alteration is localized to the infected cells, we also reasoned that the apical domain fraction of hepatocytes would not be altered upon salicylate treatment, i.e., the perturbation would specifically affect the PVM-BC association. In order to assess this, we analyzed the voxels of apical domains juxtaposed with the surface of hepatocytes, as well as with the PVM, in sections from infected animals treated or not with salicylate. The results show that salicylate treatment does not result in a relative alteration of the apical domain organization on the hepatocyte surface in infected or uninfected cells (Figure 4D). In contrast, salicylate treatment significantly decreases the contact of the apical domain with the PVM at 33 hpi, as shown by the reduced surface voxels of CD13 on the PVM (Figure 4E), indicating that the contact between the apical domain and the PVM is decreased during AMPK activation. In view of these results, we propose that the salicylate-mediated reduction in the contact of the PVM with the hepatocyte apical domain contributes to the arrest of parasite growth *in vivo*.

## Discussion

**Figure 5:**
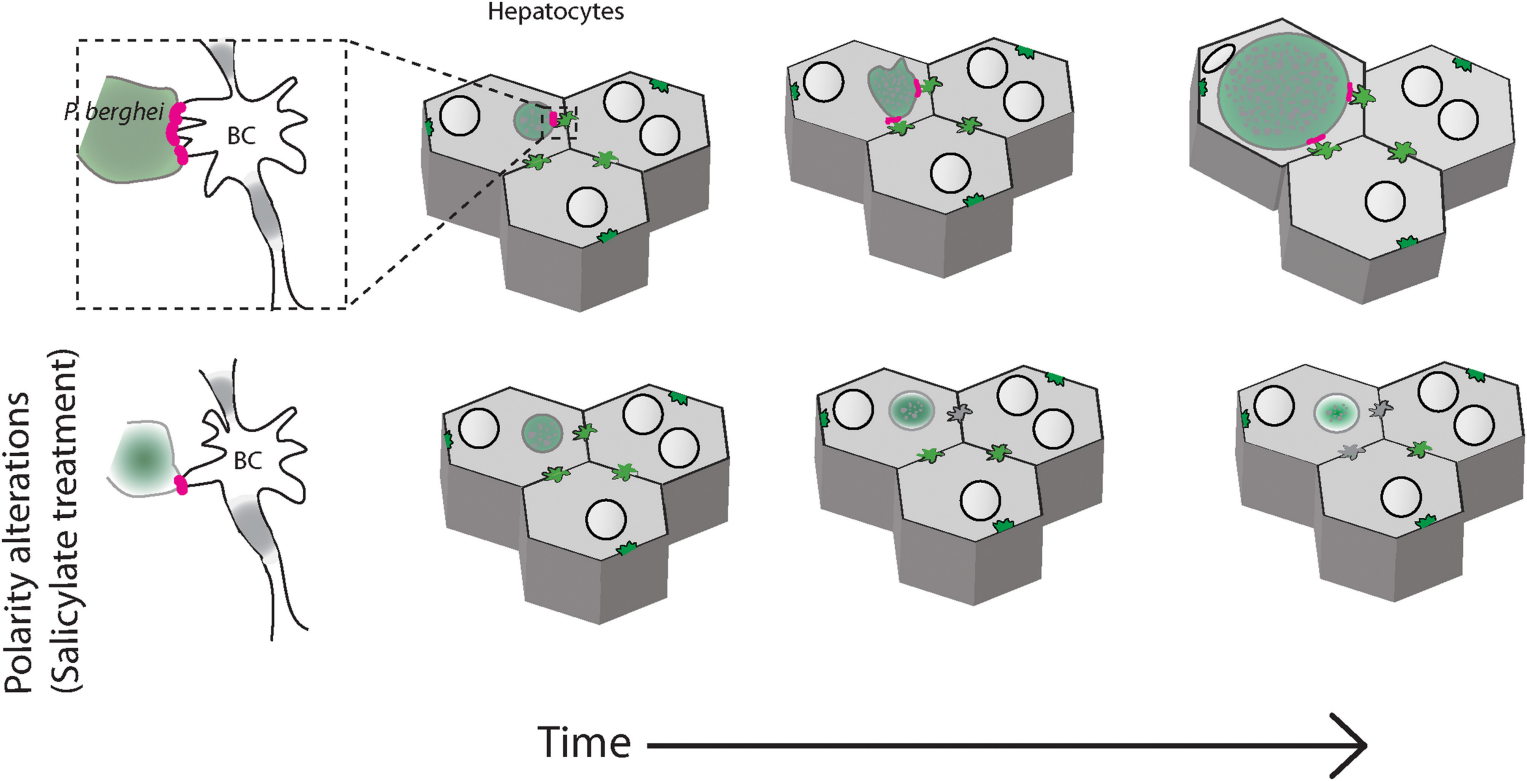
Schematic of *P. berghei* interacting with the apical domains of hepatocytes during liver stage development in the liver tissue. *Plasmodium berghei* (green) development within hepatocytes (gray) requires interaction of the parasite vacuole membrane with the hepatocyte apical domain, or bile canaliculi (BC). When hepatocyte polarity is altered by salicylate treatment, the interaction is reduced and the parasite growth is arrested (bottom panels).

The data presented in this manuscript suggests that in infected liver tissues, *Plasmodium* parasites associate with the apical domain of hepatocytes and influence the bile canalicular geometry around the infected cells, alterations that may impact liver function. Modulation of bile canalicular organization by pharmacological activation of AMPK results in abrogation of the localized PVM-BC apposition, and correlate with a significant reduction in the parasite development (Figure 5). Our results provide the first report of the involvement of the hepatocyte apical domain and bile canaliculi organization, crucial components of hepatocyte polarity, in the development of *P. berghei* parasites. Further studies will be needed to confirm these findings in human *Plasmodium* infections.

Hepatocytes have a complex and unique polarity that is central to their function (Elias, 1949; Arias et al., 1993; Treyer and Musch, 2013; Musch, 2014; Gissen and Arias, 2015). A major component of hepatocyte polarity is the organization of the apical domains. Our EM observations show a preferential association of PVM with the apical domains at 48 hpi. These results are further confirmed by high resolution fluorescence imaging in 3D at 48 hpi, as well as at earlier time points where the parasite size is not a confounding factor. Our analysis in 3D using two independent methods validate the preferential association of PVM with the apical domains of the hepatocyte. In order to establish a functional link for this association, further studies aimed at detecting features of functional contact sites such as tethering or lack of fusion, or at identifying the proteome/lipidome of the contact site are required.

It is well-known that the hepatocyte's apical domain has higher cholesterol, phospholipids and sphingomyelin content than the basolateral membrane (Meier et al., 1984; Godoy et al., 2013). Apical membranes are also highly convoluted, hence offering more membrane surface per unit volume. Hence it is tempting to speculate that the apical membrane is a potential source for membranes/lipids employed by the parasite during its rapid expansion phase in the liver. Additionally, apposition with the apical domain could provide the parasite with access to the extracellular environment and, possibly, to additional sources of nutrients. Indeed, phosphatidylcholine, one of the major phospholipids required for parasite survival in liver (Itoe et al., 2014), is actively transported to the bile canaliculi through the transporters present specifically in the apical domains (Gissen and Arias, 2015). Alternatively, the parasite could manipulate the trafficking of specific transporters such as an apical domain bound PC transporter to access such polarized nutrients. A comprehensive characterization of host proteins on the PVM will be important to address such questions.

The gallbladder is a crucial component of the entero-hepatic system which stores the biliary secretions from liver. Hepatocyte polarity is central to the biliary secretion of liver, since bile acids are absorbed from the sinusoids through the basal side of hepatocytes and secreted into the bile canaliculi on the apical side (Reshetnyak, 2013; Gissen and Arias, 2015). The etiology of the increased gall bladder bile acid levels during the liver stage of *Plasmodium* infection is therefore complex. Alterations in bile canalicular geometry could influence BC contractions and bile acid transport activity, which, in turn influence bile transport and clearance to the gall bladder (Layden and Boyer, 1978; Baumgartner et al., 1986, 1987; Morales-Navarrete et al., 2015; Sharanek et al., 2016). Pharmacological modulation of bile canalicular geometry indeed alters the bile flow/velocity along the central vein—portal vein axis (Meyer et al., 2017). In support of this, alterations in bile canalicular geometry and contractility during drug-induced cholestasis (Sharanek et al., 2016) correlate with increased levels of bile acids (Fattinger et al., 2001), while, in turn, bile acids promote hepatocyte polarity (Fu et al., 2010, 2011a,b). The dose dependency of bile acid levels with increasing parasite load suggests a possible cumulative effect resulting from several localized alterations around the sites of individual infected cells. Detailed investigations on the localization of different bile acid transporters during Plasmodium infections could shed further light into the mechanisms involved.

Pharmacological manipulation of bile canalicular geometry by well-tolerated drugs like salicylate, as reported here, opens up the possibility of controlled manipulation of polarity. The effect of AMPK on polarity is documented in different contexts, including Drosophila, neurons and polarized cell lines (Shackelford and Shaw, 2009). In a collagen sandwich model for polarized hepatocytes, AMPK activation results in acceleration of bile canalicular formation (Fu et al., 2010), through mechanisms that are not well-characterized (Gissen and Arias, 2015). Here, we show that in adult liver tissues, salicylate, a well-known AMPK modulator, alters the geometry of the bile canaliculi, with a concomitant decrease in the BC association with the PVM and the arrest of parasite growth. However, potential off-target and pleiotropic effects of salicylate cannot be ruled out entirely. It will be important to evaluate whether other well-known AMPK activators, including widely used drugs like metformin, show similar effects on AMPK modulation, hepatocyte polarity, and parasite development *in vivo*.

The idea of targeting the host to tackle infectious diseases, including malaria, has gained significant traction in recent years (Collier et al., 2013; Zumla et al., 2016; Glennon et al., 2018). Our results provide an example of a host process that is amenable to therapeutic intervention against malaria. Pharmacological modulation of hepatocyte polarity by well-tolerated widely used drugs might fast track approaches to host-directed prophylactic therapeutics. Hepatic dysfunction is strongly associated with malaria (Reuling et al., 2018), with a significant number of malaria patients showing impaired liver functions including hyperbilirubinemia, and jaundice (Joshi et al., 1986; Kaeley et al., 2017). While the pathological basis of hepatic dysfunction during malaria is not clear, drug-induced hepatotoxicity and clearance of infected RBC's have been proposed to play major roles in hepatic pathology (Reuling et al., 2018). Our results suggest that primary modulation of hepatocyte function during liver stage infection could have a direct but subtle effect on liver function. The contribution of these liver stage-specific alterations to the overall hepatic dysfunction observed during malaria remains to be determined.

## Data Availability Statement

The datasets generated for this study are available on request to the corresponding author.

## Ethics Statement

The animal study was reviewed and approved by Animal ethics committee at Instituto de Medicina Molecular, Lisbon.

## Author Contributions

LB, TS, SD, and KS were responsible for image analysis algorithms and performing analysis. VZ-L, VS, DM, AA, HR-P, and CR worked on animal infections, experimentation, image acquisition, and analysis. DM, TF, and JM worked on electron microscopy, sample preparation, and imaging. MP, MM, VZ-L, and VS conceptualized the study and edited the manuscript. VS was responsible for the study direction, funding, and manuscript draft.

### Conflict of Interest

The authors declare that the research was conducted in the absence of any commercial or financial relationships that could be construed as a potential conflict of interest. The reviewer GF declared a past collaboration with one of the authors MP to the handling editor.
