# Supplementary material for: Association of Plasmodium berghei With the Apical Domain of Hepatocytes Is Necessary for the Parasite's Liver Stage Development
Source: Front Cell Infect Microbiol. 2020 Jan 17;9:451. doi: 10.3389/fcimb.2019.00451 (PMC6978659; doi:10.3389/fcimb.2019.00451)
Supplement: Supplementary file 4 [file Data_Sheet_1.pdf]

Supplementary materials and figures

Association of *Plasmodium berghei* With the Apical Domain of Hepatocytes Is Necessary for the Parasite's Liver Stage Development

Lakshmi Balasubramanian[1], Vanessa Zuzarte-Luís[2], Tabish Syed[3], Debakshi Mullick[1], Saptarathi Deb[1], Harish Ranga-Prasad[1], Jana Meissner[4], Ana Almeida[2], Tobias Furstenhaupt[4], Kaleem Siddiqi[3], Miguel Prudêncio[2], Cecilia Rodrigues[5], Maria Mota[2] and Varadharajan Sundaramurthy[1]*

[1] National Center for Biological Sciences, Bangalore, India
[2] Instituto de Medicina Molecular, Faculdade de Medicina, Universidade de Lisboa, Lisbon, Portugal
[3] School of Computer Science and Centre for Intelligent Machines, McGill University, Montreal, QC, Canada
[4] Max Planck Institute of Molecular Cell Biology and Genetics, Dresden, Germany
[5] Research Institute for Medicines, University of Lisbon, Lisbon, Portugal
*varadha@ncbs.res.in

Image analysis workflows

The overview of the workflow for image analysis used in the manuscript is shown in Figure S1. The representative images used for the segmentation are shown in Movie S1A i, ii. The details of the Fiji macros used in the semi-automated analysis are described below.

Semi-automated intensity based method for segmentation of Bile canaliculi (BC) and Parasitophorous Vacuolar Membrane (PVM)
The four channel z-stack confocal images were split and the channels for nuclei (DAPI), PVM (UIS4), Bile canaliculi (CD13) and actin (Phalloidin) were processed separately for segmentation. The smoothed BC channel was thresholded and segmented using the intensity of CD13. The segmented output was saved as a binary mask after post processing steps including dilation, erosion and hole filling. From the resulting mask, 3D features were extracted using the ImageJ plugin, 3D object counter. The smoothed PVM was segmented manually in individual Z-stacks using Segmentation Editor (https://imagej.net/Segmentation_Editor). This plugin in Fiji allows the user to edit the segmentation that is being obtained. The edited segmentation from planes of the Z-stack is further reconstructed in 3D. The segmented PVM was also saved as a binary mask and 3D features were computed. The workflow and exact segmentation steps are mentioned in Fig S1 and macro available in supplementary.

Segmentation of Hepatocytes
Phalloidin staining is used to segment the hepatocyte cell boundary (Morales-Navarrete et al., 2015). First, the phalloidin channel image is processed using 3D median filtering, with a sigma cutoff of 2, to remove outliers. The filtered Phalloidin channel is then used to segment the hepatocytes using Segmentation Editor (as mentioned in previous section) in Fiji. The segmented hepatocytes (infected and uninfected) masks are saved and used to extract features in 3D. The workflow and segmentation steps are illustrated in Fig S1.

Calculation of the surface area of Bile canaliculi on the PVM/hepatocyte
To estimate the regions of Bile Canaliculi (BC) close to the PVM/hepatocyte, we calculate the intersection of the segmented BC mask with a dilated PVM/hepatocyte segmentation mask. For our analysis we empirically chose the dilation radius to be 5 voxels. The calculation of the surface voxels of BC on PVM/hepatocytes involves two steps. First the segmented binary mask of BC is subtracted from the expanded PVM /hepatocytes, which results in a mask with small missing portions of the PVM/hepatocyte region, as shown in Figure S2. The resultant mask is then subtracted from the mask of the expanded PVM /hepatocytes. This results in a mask of the Bile Canaliculi that are on the PVM/hepatocytes. The number of voxels in the intersection region, counted using the ImageJ 3D object counter, was used as a proxy for the surface area of BC in contact with the PVM. This measurement is a part of the workflow that is shown in Fig S1 as estimation of BC on PVM and infected/uninfected hepatocytes.

Quantification of Bile canaliculi contacts with the PVM in 2D (Using CellProfiler)
Apart from calculating the surface voxels of the BC close to the PVM, we also compute a descriptor for weighted surface area, weighted by the BC channel intensity. We first compute the intersection between the BC and the dilated PVM, and then sum the intersection voxels weighted by the corresponding intensity value in the BC channel. This calculation was done in 2D using Cell profiler, as shown in Figure S3, and was repeated for every slice of a stack.

The final value for this descriptor is calculated by summing the value from each z-slice. The CellProfiler pipeline is provided as a supplementary file.

Calculation of 'tubeness' and related network features for the Bile canaliculi
In order to evaluate the geometric properties of the BC network, we compiled a macro in Fiji. This macro measures the branch length, branch points, average branch length and diameter of the BC network. The BC channel is segmented by intensity based thresholding after a smoothening step. The workflow is illustrated in Figure S4. The parameters that are considered for pre and post segmentation is described below. The Fiji macro is provided as a supplementary file.

Initialization of parameters

The list of parameters are as follows:

i. Bile radius for closure (to specify the approximate radius of the BC): This value is used to fill holes in the BC after segmentation.
ii. Sigma radius for tubeness: This value is used for Gaussian pre-filtering.
iii. Minimum tube volume (in voxels)
iv. Remove branches with end-points (True/False): This is the method used for skeleton pruning.
v. Dilate skeleton for viewing by (voxels): This value is used to dilate the resulting skeleton for 3D visualisation.

3D Morphological closing of tubular structures
We used gray scale morphological closing (Vincent, 1993) using a spherical structural element to close hollow structures, which facilitates the segmentation of the BC as a tubular structure. This is implemented by selecting the CloseGray filter in Plugins>3D>3D Fast Filters, with the same kernel radius used in all dimensions. The approximate radius of the BC is used as the closing radius for the tubes.

Before the actual tubeness calculation, a Gaussian filter is applied to remove local noise from the filamentous voxels. This Gaussian prefiltering indirectly determines the size of the neighborhood taken into account for computation of the local intensity distribution. A good criterion to determine whether a voxel is a part of a filament is to check if there is one direction along which the intensity is constant and two perpendicular directions along which the intensities quickly drop. The tubeness (Plugins>Analyze>Tubeness) calculation computes a metric reflecting to what extent a voxel and its neighborhood fulfill this criterion (Sato et al., 1998).

Segmentation of tubular structures
In order to analyze the tubular structures, it is important to segment the voxels of the BC from the background. This is done using the Image>Adjust>Threshold function. During the process of thresholding, small objects that are not connected to the rest of the neighborhood can get segmented. Such small objects are removed using Analyze>3D Objects Counter. The resulting output is saved.

Skeletonization and Analysis of the Tubular Network
This macro is used to characterize the network features of the BC, including branch length and branch points. We first reduce the thickness of the segmented BC tubes to their one voxel wide centerlines. This process of skeletonization is carried out using

Plugins>Skeleton>Skeletonize (2D/3D). In order to analyze and identify the branch and end points of the skeletonized network, we used the following criteria,

i. End-point voxels have less than two neighbors.
ii. Junction voxels have more than two neighbors.
iii. Slab voxels (remaining voxels) have exactly two neighbors.

For better visualization, the resulting skeleton in 3D is dilated and saved as a tiff image.

Skeleton Pruning

Before calculating the skeleton features we remove any short branches from the skeleton. The extraneous branches, which arise due to non-uniformity in the boundary of the segmentation mask, were removed by pruning the skeleton. We use end-point pruning using Plugins>Skeleton>Analyze Skeleton (2D/3D).

Extraction of relevant parameters

A number of network features were computed form the skeleton and the BC segmentation mask using ImageJ plugins as listed below.

i. Total length, l of BC skeleton: The total length of the BC is computed as summation of the average branch length to number of branches over all slices in a Z-stack obtained using the ImageJ plugin "Analyze skeleton".
ii. Number of branch points: This is the summation of the branches over all slices in a Z-stack.
iii. Total volume, v of BC mask: $Number\ of\ BC\ voxels * volume\ of\ 1\ voxel$
iv. Mean cross sectional area, $a = \frac{v}{l}$ of BC mask
v. Mean Diameter, d of BC $2 * \sqrt{\frac{a}{\pi}}$

Automated flow-based method to segment the Parasitophorous Vacuolar Membrane (PVM) and the Bile canaliculi (BC)

The same images (PVM (UIS4) and Bile canaliculi (CD13) channels) that were used for intensity-based segmentation were also used for flow-based segmentation (Vasilevskiy and Siddiqi, 2002; Boykov and Funka-Lea, 2006) for all the three time points of infection. This method is based on the formulation and derivation of a flux maximizing geometric flow, which has been previously applied to the segmentation of tubular structures, such as blood vessels. Since it is a flow-based method in all the directions from the initial seed points, it not only considers the magnitude but also the direction of an appropriate vector field. We place seeds within the interior of the network; these seed positions are automatically selected using a notion of flux derived from the CD13 channel. These seeds then flow outwards, following a partial differential equation so as to maximize the flux of the gradient of the original intensity image through the evolving surface. The evolving surface is the collection of all dilating seeds. The seeds merge as they reconstruct parts of the network. The flow stabilizes as soon as it touches the boundary of the bile duct from within, and eventually it covers the entire connected pieces of the network to segment them. The flow can handle an arbitrary number of seeds and networks of arbitrary complexity. An example of flow-based segmentation of the BC is provided in the movie Mov S4B i, ii. Network features of the segmented BC are calculated using Fiji. To segment the PVM, initial seeds are placed outside the PVM structure in the PVM channel. An example of flow-based segmentation of the PVM is provided in the movie Mov S1C. A 3D view of the segmented PVM at the three time points is shown in the movie Mov S1D. A direct comparison between threshold based and flow based segmentation for BC (Fig 4A, B) and PVM (Fig 1C, D) shows a strong agreement between the two methods.

Assessing the specificity of the PVM-BC interaction in 3D using flow based analysis
All subject data are the 24 and 33 hpi time points. The analysis was not feasible for the 48 hpi time point data because the large size of the PVM would limit the use of such an analysis.

First, we computed an outward Euclidean distance function from the boundary of the BC network, to all points in the volume. We did this by implementing an outward grassfire flow (an outward evolving surface with unit speed in the direction normal to the initial BC network surface). This process yields the distance from any point in the volume outside the BC network to the closest point on the network. By thresholding this distance function at any chosen value, we have the locations of all points in the volume which are at that distance, such that sphere drawn with its radius equal to that value would just touch the BC network. (Figure S6). In the figure we also show locations that are closer than "r" to the BC network in yellow, locations that are at a distance between "r" and "r + delta" to the BC network in blue, and locations that are at a distance greater than "r + delta" to the BC network in grey. Using this construction, we calculated the volume of intersection of spheres of radius "r" equivalent to the volume of the PVM (i.e. $4/3\ \pi r^3$) with the BC network. This corresponds to the blue region (Figure S6). We also calculated the volume of locations at a distance greater than "r + delta", i.e., the grey region in Figure S5. In our analysis to define the band around the sphere we chose a delta of 5 voxels, so as to not artificially reduce the probability of intersection. Averaged over all time points and over all subjects, we found that the blue and grey regions have the following relative volumes: Time point 24hpi: blue region percentage of touching voxels = 4.03%; grey region percentage of not touching voxels = 4.19%. Time point 33hpi: blue region percentage of touching voxels = 1.52%; grey region percentage of not touching voxels = 2.97%. Taken together, this shows that if randomly placed at a distance "r" from the BC network an inert bead with volume $4/3\ \pi r^3$ equal to the volume of the PVM would be more likely to not intersect the BC network than intersect it. Thus, the observed apposition of the PVM with the BC network in our experiments is not accidental, i.e., beads with a similar size are more likely to be found at locations away from the BC network.

References:

Boykov, Y., and Funka-Lea, G. (2006). Graph Cuts and Efficient N-D Image Segmentation. *Int J Comput Vision* 70(2), 109-131.

Morales-Navarrete, H., Segovia-Miranda, F., Klukowski, P., Meyer, K., Nonaka, H., Marsico, G., et al. (2015). A versatile pipeline for the multi-scale digital reconstruction and quantitative analysis of 3D tissue architecture. *Elife* 4. doi: 10.7554/eLife.11214.

Sato, Y., Nakajima, S., Shiraga, N., Atsumi, H., Yoshida, S., Koller, T., et al. (1998). Three-dimensional multi-scale line filter for segmentation and visualization of curvilinear structures in medical images. *Med Image Anal* 2(2), 143-168.

Setchell, K. D., Rodrigues, C. M., Clerici, C., Solinas, A., Morelli, A.,Gartung, C., et al. (1997). Bile acid concentrations in human and rat liver tissue and in hepatocyte nuclei. Gastroenterology 112, 226–235.

Vasilevskiy, A., and Siddiqi, K. (2002). Flux-Maximizing Geometric Flows. *IEEE Transactions on Pattern Analysis and Machine Intelligence* 24(12), 1565-1578.

Vincent, L. (1993). Morphological grayscale reconstruction in image analysis: applications and efficient algorithms. *IEEE Trans Image Process* 2(2), 176-201. doi: 10.1109/83.217222.

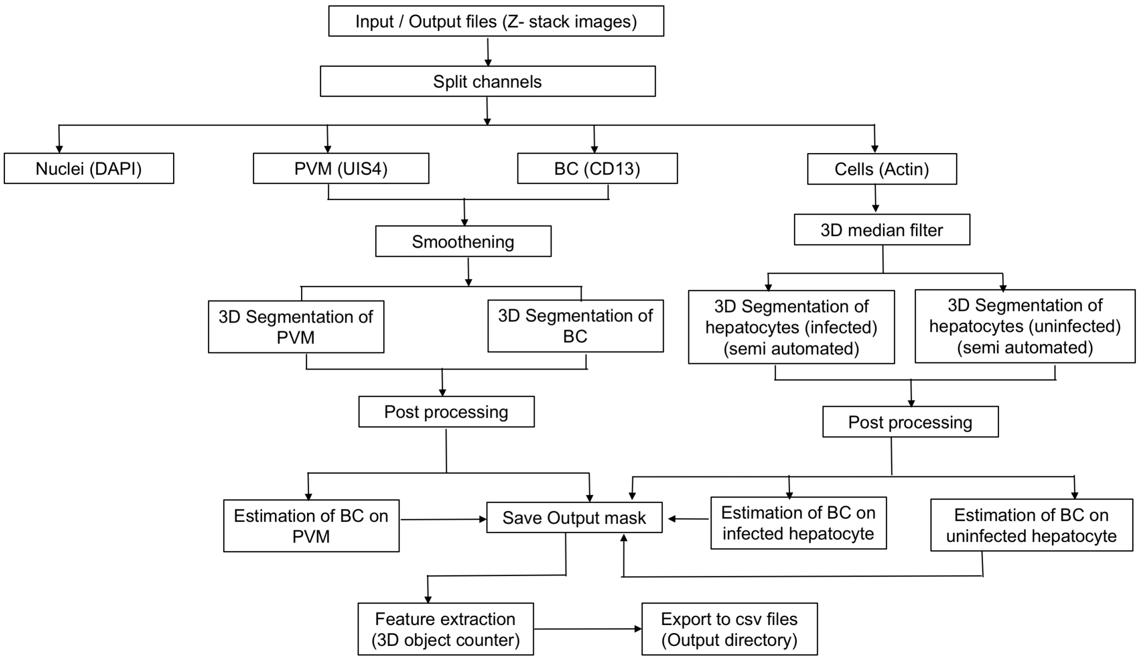

Figure S1. A schematic of the image analysis workflow for the semi-automatic intensity based segmentation of the Bile Canaliculi (BC), the PVM, and the infected and uninfected hepatocytes in 3D.

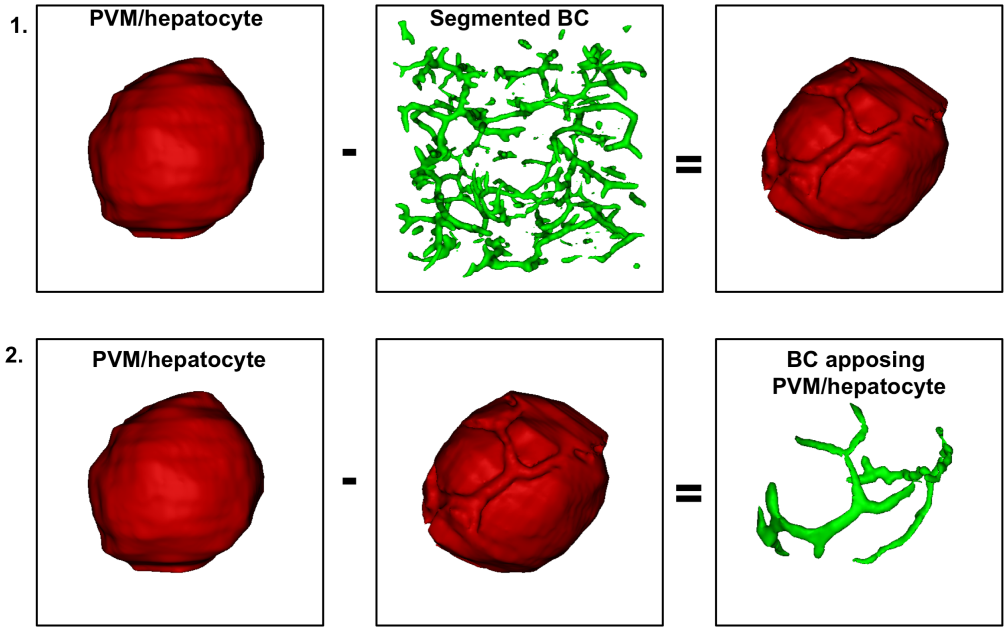

Figure S2. Quantification of the surface voxels of the Bile canaliculi (BC) on the PVM/hepatocyte. 1. The segmented BC mask is subtracted from the PVM/hepatocyte mask. 2. The result obtained from step 1 is subtracted from the PVM/hepatocyte mask, which results in extraction of the BC region(s) within a specified distance from PVM or hepatocyte.

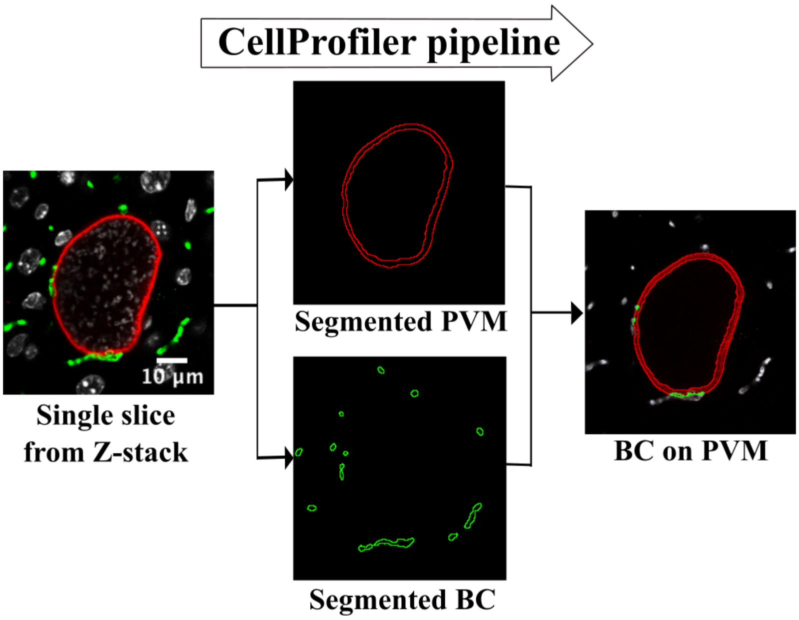

Figure S3. A schematic of the CellProfiler pipeline used to extract the Bile Canaliculi (BC) in close proximity to the PVM in 2D.

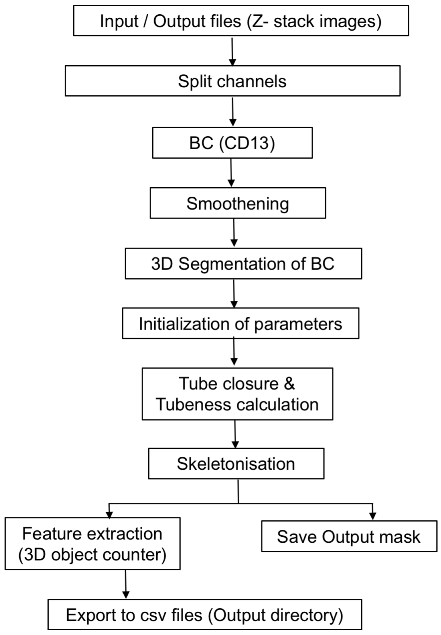

Figure S4. A schematic of the image analysis workflow for the estimation of tubeness and network features of the segmented Bile Canaliculi (BC).

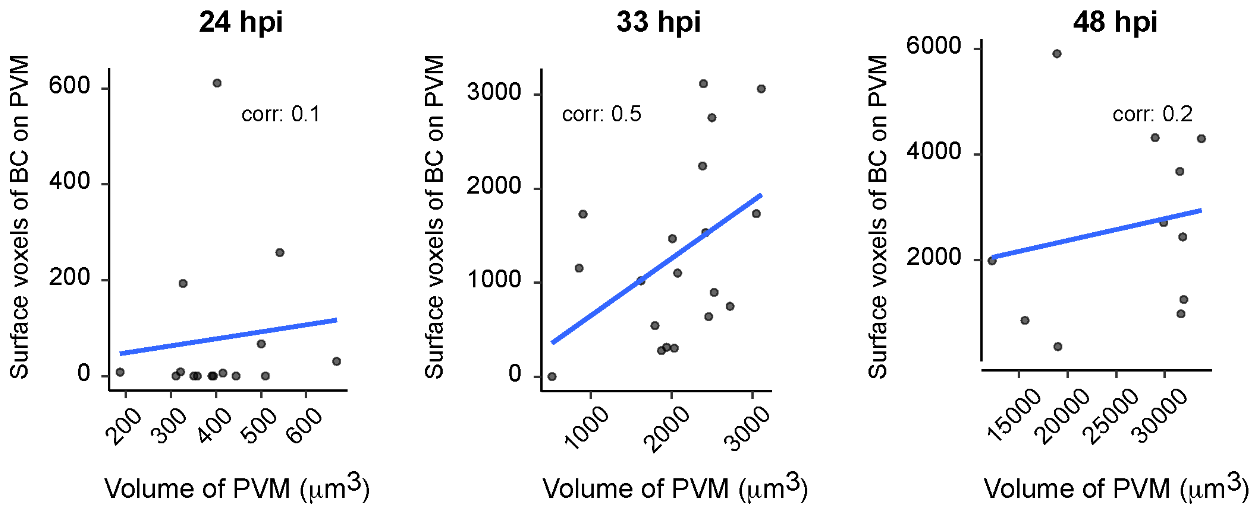

Figure S5. Correlation analysis between the PVM volume and surface voxels of BC on PVM at different time points post infection.

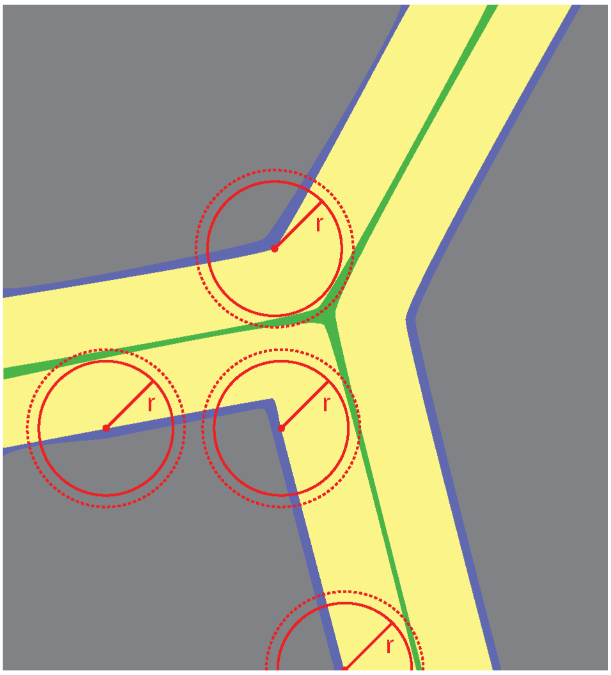

Figure S6. Assessing the specificity of association between PVM and BC using flow based segmentation. For illustration, a portion of the BC network is shown in green, and the centres of selected spheres at a distance ‘r’ from the BC network shown in red, with slightly dilated sphere shown in dotted red color. The yellow region denotes all locations closer than radius ‘r’ from the BC network. The grey region indicates all locations that are greater than ‘r+delta’ to the BC network. The blue region indicates a band that are within a distance ‘r’ and ‘r+delta’ in distance to the BC network. Whereas this schematic is in 2D, the actual analysis is done in 3D.

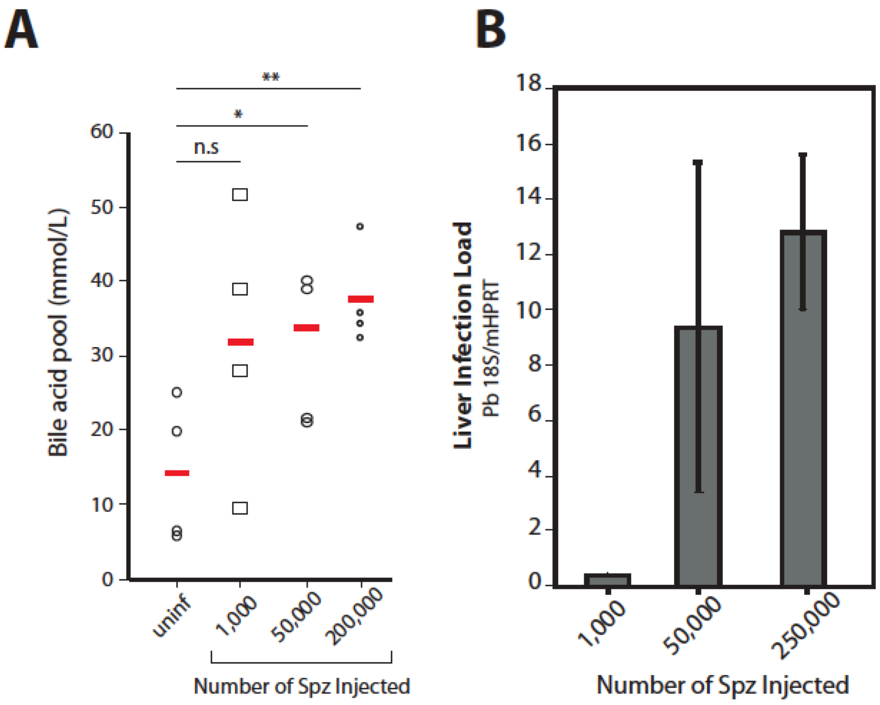

Figure S7. Liver parasite load dependence of bile acid levels
(A) Total bile acid levels in gallbladders of mice infected with increasing number of sporozoites, assessed 40 hours post infection. Bile acids were determined using gas chromatography following liquid-solid extraction (Setchell et al., 1997). Data point denotes individual mice. Significance was assessed by Student's *t*-test, n.s denotes not statistically significant, * and ** denotes p values < 0.05 and < 0.01 respectively.
(B) Parasite load in liver from mice infected with the indicated number of sporozoites. Error bars represent standard deviation of mean from 4 mice for each condition.

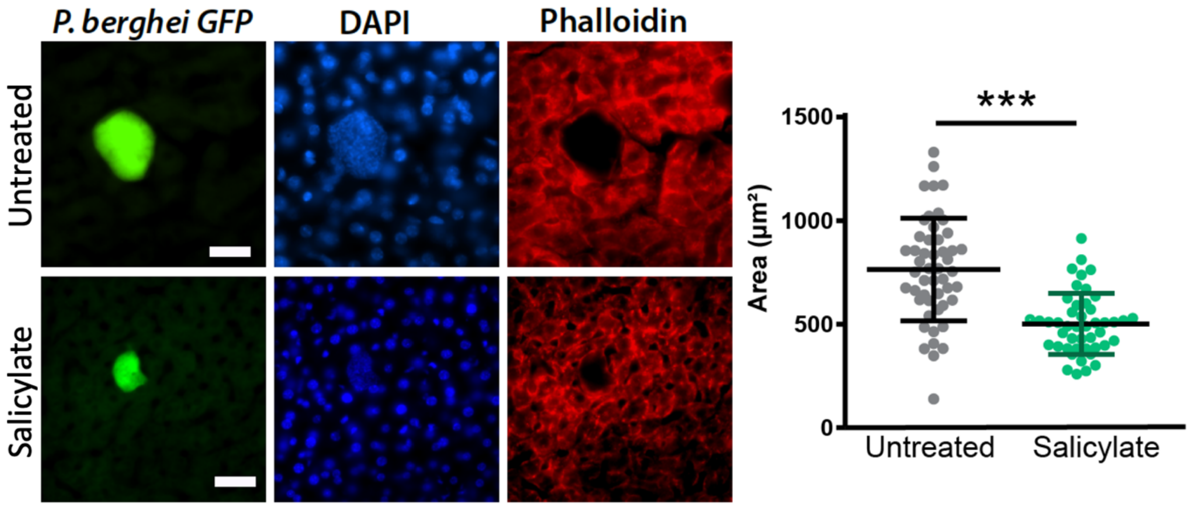

Figure S8. Salicylate treatment impairs the development of *P. berghei in vivo*. Mice were infected with *P. berghei* expressing GFP and treated with Salicylate. 48 hours post infection, sections from control and Salicylate treated mice were imaged and area of the parasite quantified. *** denotes p value of < 0.001 based on Student's *t*-test, scale bar is 10 µm. Result is representative of two biological experiments.
